# Circulating vascular endothelial growth factor and cancer risk: A bidirectional mendelian randomization

**DOI:** 10.3389/fgene.2022.981032

**Published:** 2022-09-07

**Authors:** Hong Wu, Tianjun Ma, Dongli Li, Mei He, Hui Wang, Ying Cui

**Affiliations:** ^1^ Department of Research, Guangxi Medical University Cancer Hospital, Nanning, Guangxi, China; ^2^ Department of Bone and Joint Surgery, The First Affiliated Hospital of Guangxi Medical University, Nanning, Guangxi, China; ^3^ Department of Orthopaedic Trauma and Hand Surgery, The First Affiliated Hospital of Guangxi Medical University, Nanning, Guangxi, China

**Keywords:** VEGF, cancer, GWAS, SNP, mendelian randomization

## Abstract

In observational studies, circulating vascular endothelial growth factor (VEGF) has been reported to be associated with certain types of cancer. The purpose of this study was to verify whether there is a causal relationship between circulating VEGF and different types of cancer and the direction of the causal relationship. Summary statistical data were obtained from the corresponding genome-wide association studies (GWASs) to investigate the causal relationship between circulating VEGF and the risk of several cancers, including breast cancer, ovarian cancer, lung cancer, colorectal cancer, anus and anal canal cancer, prostate cancer, esophageal cancer, kidney cancer, bladder cancer, thyroid cancer, malignant neoplasm of the brain and malignant neoplasm of the liver and intrahepatic bile ducts. A two-sample bidirectional Mendelian randomization (MR) analysis and sensitivity tests were used to evaluate the validity of causality. A causal relationship was detected between circulating VEGF and colorectal cancer (OR 1.21, 95% CI 1.11–1.32, *p* < 0.000) and colon adenocarcinoma (OR 1.245, 95% CI 1.10–1.412, *p* < 0.000). Suggestive evidence of association was detected in VEGF on malignant neoplasms of the rectum (OR 1.16, 95% CI 1.00–1.34, *p* = 0.049). No causal relationship was found between circulating VEGF and other types of cancer, nor was there a reverse causal relationship from tumors to VEGF (*p* > 0.05). Circulating VEGF has a causal relationship with specific types of cancer. Our findings highlight and confirm the importance of circulating VEGF in the prevention and treatment of colorectal cancer.

## Introduction

The genesis and development of malignant tumors depend on angiogenesis. Tumor angiogenesis is a complex process that is regulated by both angiogenic and angiosuppressive factors (Folkman and Kalluri, 2004). Neovascularization increases the blood supply to the tumor to better deliver oxygen and nutrients, allowing tumor cells to spread to distant sites (Verheul et al., 2004). Among the proangiogenic factors, vascular endothelial growth factor (VEGF) is probably the most important (Eichholz et al., 2010). The VEGF-associated gene family for angiogenesis includes five glycoproteins, named VEGF-A, VEGF-B, VEGF-C, VEGF-D, VEGF-E, and two placental growth factors (PLGF-1 and -2) (Ferrara et al., 2003). VEGF-A, commonly known as VEGF, is a vascular permeability factor and plays a key role in normal physiological and pathological angiogenesis. VEGF is mainly produced by perivascular cells and can also be produced by tumor cells. It acts on endothelial cells through a paracrine mechanism to promote vascular formation, inhibit endothelial cell apoptosis, and provide vascular permeability (Grothey and Galanis, 2009). Observational experiments have shown that VEGF is overexpressed in most human tumors and is closely related to the growth, metastasis, pathological grade and poor prognosis of gallbladder carcinoma (Jiang et al., 2018; Xu et al., 2019), esophagogastric cancer (Gray et al., 2012), gastric cancer (Ohta et al., 2003), colorectal cancer (Fujisaki et al., 1998; Eldesoky et al., 2011), ovarian cancer (González-Palomares et al., 2017; Komatsu et al., 2017), breast cancer (Banys-Paluchowski et al., 2018), and uterine cervical cancer (Sawada et al., 2019). Therefore, VEGF is often used as a circulating marker to detect tumor occurrence and development. VEGF is also important in the switch to the angiogenic phenotype during early tumorigenesis. The transformation of tumor cells into an angiogenic phenotype is considered a marker of a malignant process (Fang et al., 2001). Since VEGF can be produced by perivascular cells and tumor cells, there are theoretically two mechanisms: the increase in circulating VEGF leads to tumorigenesis, or the occurrence of tumors secretes higher levels of circulating VEGF. Many observational experiments have found that VEGF is elevated in different tumor types, but whether there is a causal relationship between tumor and VEGF remains unclear. To verify the causal relationship between the two, Mendelian randomization analysis was used in this study.

Mendelian randomization (MR) is a promising causal inference tool that has emerged in recent years. The MR study design follows Mendelian inheritance rules of “parental alleles are randomly assigned to offspring”. Since genotype determines phenotype, and genotype is associated with disease through phenotype, genotype can be used as an instrumental variable (IV) to infer the association between phenotype and disease. This approach is unaffected by confounding factors and reverse causal associations, which are the reverse chronological order of exposure and outcome found in traditional epidemiological studies (Smith and Ebrahim, 2003). MR analysis needs to meet three important prerequisites: 1. Ivs are robustly associated with the exposure; 2. IVs share no common cause with the outcome; 3. IVs do not affect the outcome except through the risk factor (Davies et al., 2018). Only when the above three conditions are simultaneously met at can it be shown that the genotype is phenotypically mediated in the disease, that is, the phenotype or exposure is inferred to be the cause.

In this study, we performed a two-sample bidirectional MR using publicly available genome-wide association study (GWAS) summary statistics to explore the causal relationship between VEGF and the risk of multiple cancers, including breast cancer, ovarian cancer, lung cancer, colorectal carcinoma, prostate cancer, esophageal cancer, kidney cancer, bladder cancer, brain malignant tumors, and malignant tumors of the liver and intrahepatic bile duct. A clear causal relationship between VEGF and cancers will help prevent and treat these diseases.

## Materials and methods

### GWAS statistics of vascular endothelial growth factor

Summary statistics for circulating VEGF were retrieved from a large-scale GWAS meta-analysis of circulating cytokines and growth factors (Ahola-Olli et al., 2017). The study consists of three independent population cohorts, including the Cardiovascular Risk in Young Finns Study (YFS), FINRISK1997 and FINRISK 2002, which included 8,293 Finnish individuals in total. YFS is a multicenter follow-up study in which subjects were randomly selected from the Finnish cities of Helsinki, Kuopio and Uru and their rural areas. The study began in 1980, when 3,596 children and young adults participated in the first cross-sectional survey. Follow-up was conducted in 1983, 1986, 1989, 2001, 2007, and 2011. FINRISK surveys were cross-sectional studies conducted every 5 years to monitor the levels of chronic disease risk factors in Finland. Each survey included randomly selected subjects aged 25 to 74 from five geographical regions of Finland. The study included cytokine data from survey participants in 1997 and 2002. The GWAS statistics were adjusted for age, sex, body mass index, and the first ten genetic principal components. We included single nucleotide polymorphisms (SNPs) associated with circulating VEGF as the exposure and we also consider VEGF as an outcome in our bidirectional MR analysis. When a potential causal effect of VEGF on certain cancer was detected, we revalidated the results of it using VEGF as exposure from another GWAS (Folkersen et al., 2020). Summary statistics from the GWAS were obtained from 13 cohorts of European ancestry including 21,758 individuals with adjustment for population structure and study-specific parameters.

### GWAS statistics of different types of cancer

Summary statistics for multiple cancers were retrieved and obtained through the IEU OpenGWAS (MR Base) public database (https://gwas.mrcieu.ac.uk/). We extracted genetic variants associated with breast cancer, ovarian cancer and lung cancer from the publicly available summary statistics of the Breast Cancer Association Consortium (BCAC) (Michailidou et al., 2017), Ovarian Cancer Association Consortium (OCAC) (Phelan et al., 2017), and International Lung Cancer Consortium (ILCCO) (Wang et al., 2014). The GWAS summary statistics of colorectal cancer, anus and anal canal cancer, prostate cancer, esophageal cancer, kidney cancer, bladder cancer, thyroid cancer, malignant neoplasm of the brain and malignant neoplasm of the liver and intrahepatic bile ducts were all from the publicly available summary statistics of the FinnGen consortium (www.finbb.fi). Two-sample MR requires two independent samples from the same population, and people of European ancestry were included in this study. Specific data for different tumor types, including sample size, population, and number of single nucleotide polymorphisms (SNPs), are summarized in Supplementary Table S1. When a potential causal effect of VEGF on certain cancer (colorectal cancer) was detected, we explored the reverse causal inferring using the same MR approaches, with the cancer (colorectal cancer) as the exposure and VEGF as the outcome. This summary statistic on colorectal cancer was from a large GWAS that included whole-genome sequencing data for 1,439 colorectal cancer cases and 720 controls from five studies, and GWAS array data for 58,131 colorectal cancer or advanced adenoma cases and 67,347 controls from 45 studies from GECCO, CORECT, and CCFR (Huyghe et al., 2019). The population included was mostly European.

### Mendelian randomization statistical analysis

A two-sample bidirectional MR was used to test the causal relationship between VEGF and tumors. To verify whether circulating VEGF is a risk factor for various tumors, we first selected closely correlated SNPs from GWAS results of VEGF. In this process, VEGF acts as an exposure, and multiple types of tumors act as outcomes. To verify whether tumors lead to an increase in circulating VEGF, SNPs -related to various tumors were selected as IVs in the process of reverse MR analysis, with tumors as exposures and VEGF as an outcome.

**FIGURE 1:**
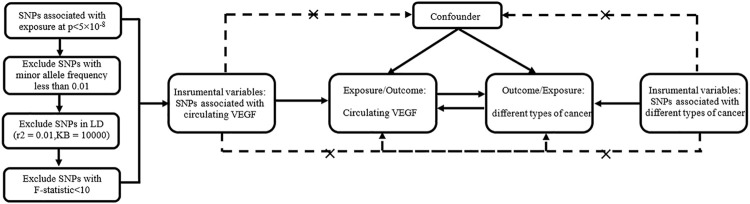
Bidirectional Mendelian randomized study design between circulating VEGF and different types of cancer.

SNPs -associated with exposure must meet the following three conditions to be selected as IVs: genome-wide significance (*p* value <5 × 10^−8^), minor allele frequency >0.01, and removal of SNPs with linkage disequilibrium (r^2^ = 0.01 and KB = 10000). The phenotypic variation explained by SNPs was calculated as follows: R^2^ = 2 × beta^2^ ×(1-EAF)× EAF/SD^2^ (SD, standard deviation), with EAF = effect allele frequency and beta = the effect of each SNP on the exposures (Shim et al., 2015). The F statistic ((N−k−1)/k) * (R^2^/(1−R^2^)) was used to test the strength of the association between these SNPs and the exposure factors, with N and k representing the sample size and number of SNPs, respectively (Burgess and Thompson, 2011). SNPs were included with strong statistical power (F statistics>10). The I^2^-GX value in MR-Egger method (Bowden et al., 2016b) was used to assess the bias of weak instrumental variables. The value should be between 0 and 1, with higher values indicating less bias. Figure 1 shows our design framework.

The Wald ratio method was used to estimate the effect of a single SNP closely related to exposure on the outcome, and the inverse variance weighting (IVW) method was then adopted to combine each SNP’s effect size. The weighted median Burgess et al. (2016) and MR–Egger (Burgess and Thompson, 2017) were used as supplements for the MR statistical analysis. Cochrane’s Q value and MR–Egger intercept (Burgess and Thompson, 2017) were used to assess the heterogeneity and horizontal pleiotropy, respectively. The MR-PRESSO (Verbanck et al., 2018) outlier test was used if there was evidence of horizontal pleiotropy. It is based on the IVW method to remove outliers and again provide an estimate of the causal effect. When heterogeneity and horizontal pleiotropy were absent, the IVW method was considered the primary assessment method. When heterogeneity existed, the multiplicative random effects IVW method results were adopted if the IVW and weighted-median results were in the same direction of causal effect. This is because the weighted median estimate will remain consistent even when up to 50% of the genetic IVs utilized are invalid, while the IVW method requires that all SNPs used as IVs be valid (Bowden et al., 2016a). Leave-one-out analysis and funnel plots were used as visual illustrations of sensitivity analysis. Leave-one-out analysis could determine whether the estimates were driven only by a single SNP. The points representing SNPs on the funnel plot were symmetrically distributed if there was no heterogeneity. The *P value* of the MR analysis results was less than the significance level of 1.28 × 10^–3^ corrected by Bonferroni (*P value* threshold = 0.05/39, corrected for 39 pairs of exposure and outcome), and the causal relationship between exposure and outcome was concluded. A *P* value between 1.28 × 10^–3^ and 0.05 was statistically significant, which was considered as suggestive evidence of association.

The statistical power of the MR analysis results was assessed by mRnd (https://cnsgenomics.shinyapps.io/mRnd/). All MR statistical analyses and data visualization were performed in R software version 4.1.1 using the “TwoSampleMR” (Hemani et al., 2018), “MR-PRESSO” (Verbanck et al., 2018) and “MendelianRandomization” (Yavorska and Burgess, 2017) packages.

## Results

The number of SNPs -associated with exposure that were extracted as instrumental variables in the bidirectional Mendelian randomization ranged from 1 to 184. Their explained variances ranged from 4.2% to 62.8%. The estimates of the variation explained by the instruments might be inflated since they were all in-sample (estimated from the discovery GWAS). The F statistics for SNPs were all greater than 10 (Table 1).

**TABLE 1:** Summary statistics of exposure.

Exposure	SNPs(n)	Sample	R^2^ (%)	F	Population	PMID
VEGF	11	7,118	15.0	104.5	European	27989323
Breast Cancer	184	228,951	29.3	515.2	European	29059683
ER+ Breast cancer	135	175,475	31.7	602.8	European	29059683
ER- Breast cancer	40	127,442	13.9	514.2	European	29059683
Ovarian cancer	12	66,450	7.2	429.5	European	28346442
High grade serous ovarian cancer	16	53,978	14.1	553.6	European	28346442
Invasive mucinous ovarian cancer	3	42,358	8.6	1328.4	European	28346442
High grade and low grade serous ovarian cancer	16	54,990	13.2	522.2	European	28346442
lung cancer	5	27,209	8.5	505.4	European	24880342
Lung adenocarcinoma	3	18,336	8.1	538.6	European	24880342
Squamous cell lung cancer	4	18,313	8.7	436.1	European	24880342
Colorectal cancer	3	218,792	4.2	3197.3	European	finnGen
Malignant neoplasm of prostate	45	95,213	62.8	3579.8	European	finnGen
Malignant neoplasm of bladder	2	218,792	7.4	8742.1	European	finnGen
Malignant neoplasm of thyroid gland	3	218,792	19.8	18004.9	European	finnGen
Malignant neoplasm of liver and intrahepatic bile ducts	1	218,792	16.5	43233.9	European	finnGen

SNPs(n) number of single nucleotide polymorphism, R^2^ phenotype variance explained by genetics, FF statistics, PMID ID of publication in PubMed.

### Causal effect of VEGF on cancer

**FIGURE 2:**
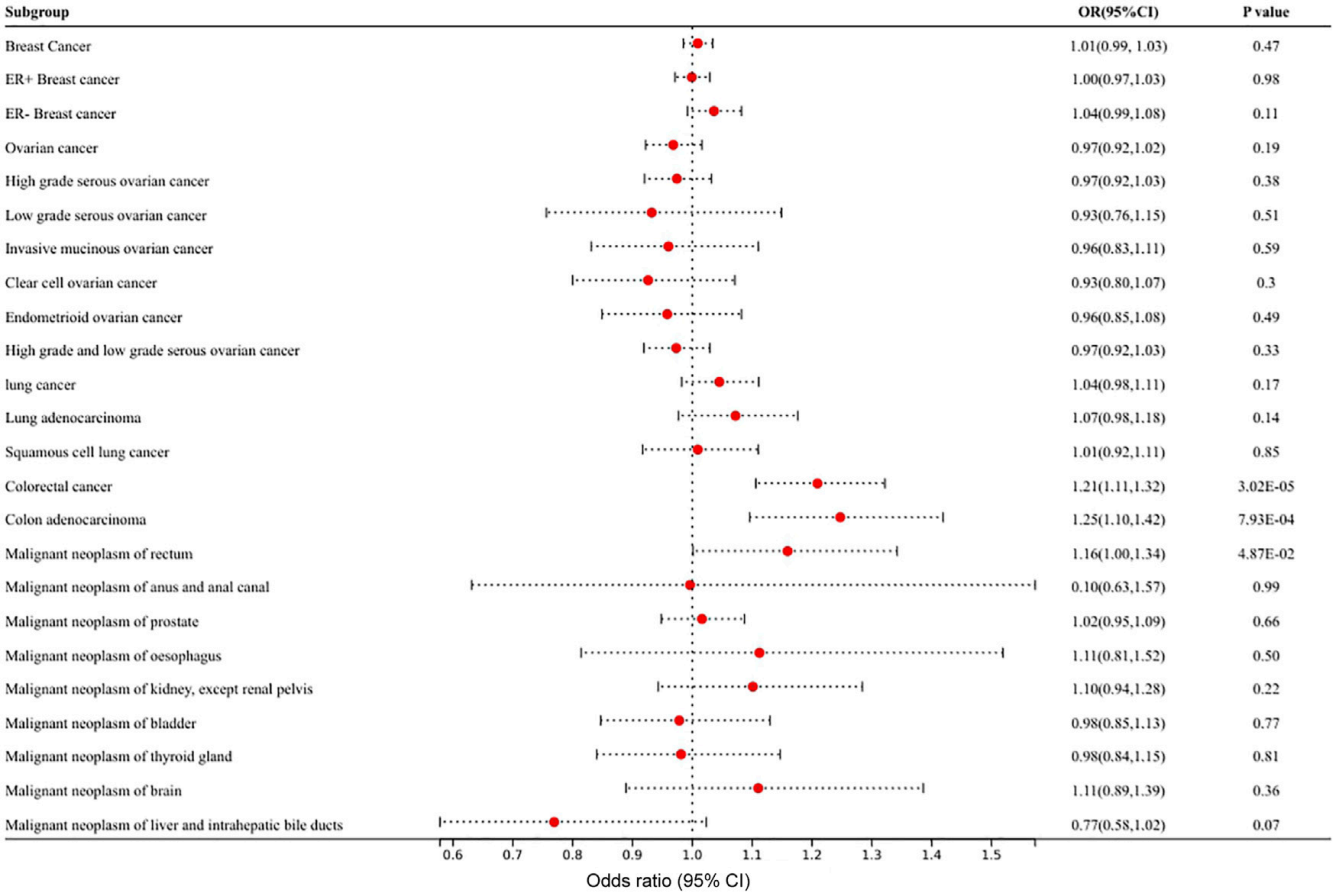
Mendelian randomization results of the association of circulating VEGF on different types of cancer.

The characteristics of 11 SNPs as IVs that were closely related to circulating VEGF are shown in Supplementary Table S2. Figure 2 and Supplementary Table S3 show the results of MR analyses of the causal effects of VEGF on different types of cancer as well as the evaluation of heterogeneity and pleiotropy effects. A causal relationship was detected in circulating VEGF in colorectal cancer (OR 1.21, 95% CI 1.11–1.32, *p* = 3.0 × 10^–5^) and colon adenocarcinoma (OR 1.25, 95% CI 1.10–1.42, *p* = 7.9 × 10^–4^) in MR analysis with the IVW method. P values were less than the significance level of 1.28 × 10^–3^ corrected by Bonferroni. High levels of circulating VEGF were associated with an increased risk of colorectal cancer and colon adenocarcinoma. Suggestive evidence of association was detected in VEGF on malignant neoplasms of the rectum (OR 1.16, 95% CI 1.00–1.34, *p* = 4.9 × 10^–2^). No causal relationship was found between circulating VEGF and other types of cancer (*p* > 0.05). Heterogeneity and horizontal pleiotropy were not observed by Cochrane’s Q value or the MR–Egger intercept method of VEGF in multiple tumors, indicating the robustness of the results of this study. A scatter plot for each pair of associations that better demonstrated causality is shown in Supplementary Figure S1; the funnel plot showed their heterogeneity (Supplementary Figure S2). The leave-one-out analysis revealed that each pair of associations was not driven by a specific SNP (Supplementary Figure S3).

The statistical power of circulating VEGF for colorectal cancer, colon adenocarcinoma, and malignant neoplasms of the rectum was 99%, 95% and 52%, respectively.

### Causal effect of cancer on VEGF

**FIGURE 3:**
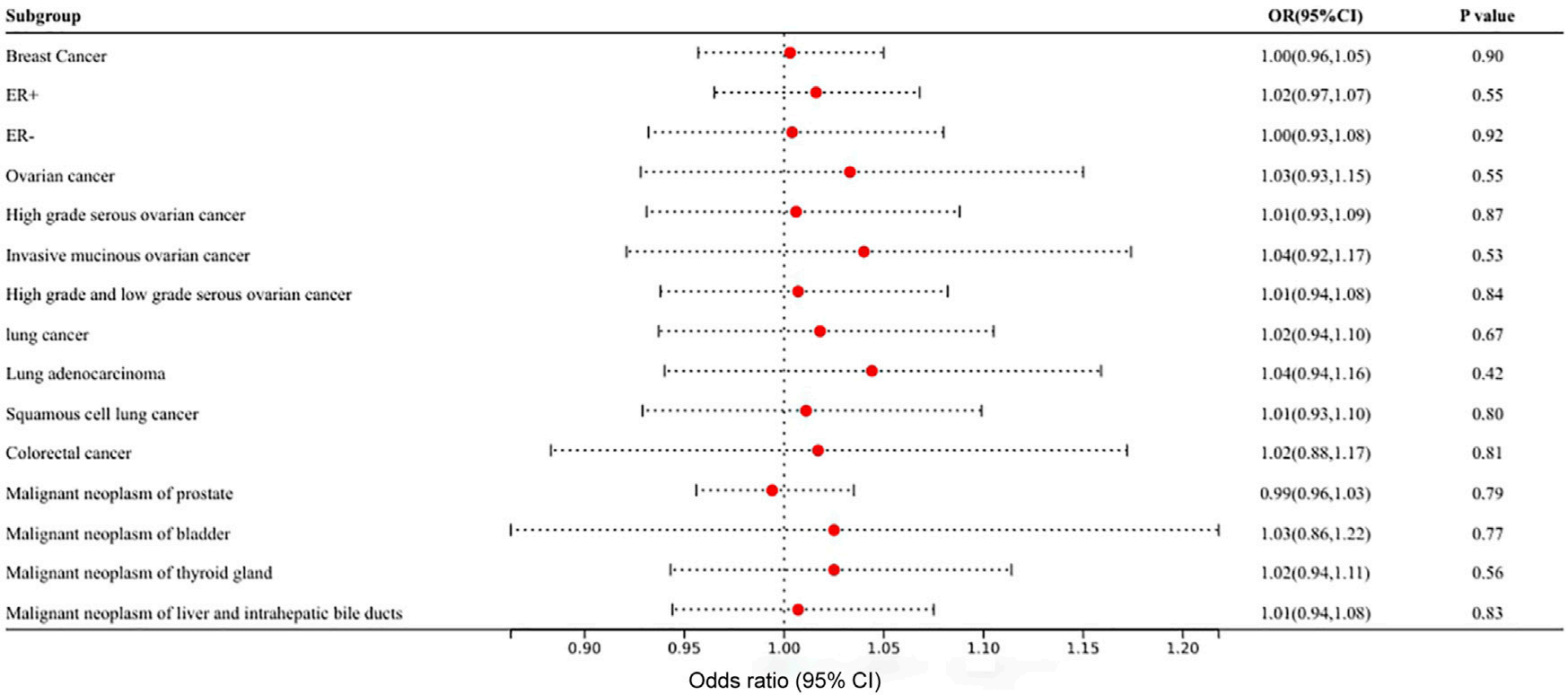
Mendelian randomization results of the association of different types of cancer on circulating VEGF.

In MR analysis of the causal relationship between different types of cancer and VEGF, strongly associated SNPs were identified in only 11 types of cancer, and the number of SNPs ranged from 1 to 156. The Wald ratio, IVW, and weighted-median methods did not detect a statistically significant causal relationship of any type of cancer on VEGF in MR analysis (*p* > 0.05). Figure 3 and Supplementary Table S4 show the results of MR analyses of the causal effects of different types of cancer on VEGF as well as the evaluation of heterogeneity and pleiotropy effects. Heterogeneity and horizontal pleiotropy were not observed by Cochrane’s Q value or the MR–Egger intercept method of multiple tumors to VEGF. Heterogeneity and pleiotropy tests were performed for at least three SNPs, a heterogeneity test was performed for only two SNPs, and a sensitivity test was not required for one SNP. A scatter plot for each pair of associations is shown in Supplementary Figure S4; the funnel plot is shown in Supplementary Figure S5. The leave-one-out analysis is shown in Supplementary Figure S6. For each pair of associations with less than three SNPs, the sensitivity analysis of the funnel plot and leave-one-out analyses was not displayed.

### Different GWAS to verify the causal effect between VEGF and colorectal cancer

**FIGURE 4:**
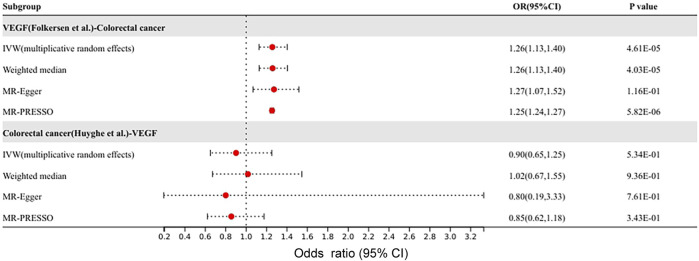
Different GWAS data to verify the causal effect between VEGF and colorectal cancer.

After the above MR analysis results, VEGF was found to have a causal effect on colorectal cancer. Since both VEGF and colon cancer subjects were from the Finnish Europeans, the overlap in sample size might have contributed to the higher false-positive results. Therefore, the GWAS data of VEGF by Folkersen et al. and colorectal cancer by Huyghe et al. were selected as instrumental variables as exposure, and MR analysis was performed again to verify the results. The results of validation were shown in Figure 4. Consistent with our previous results, the IVW method suggested a causal effect of VEGF on colorectal cancer (OR 1.26, 95% CI 1.13–1.40, *p* = 4.6 × 10^–5^). Colorectal cancer has no causal effect on VEGF (*p* > 0.05). There was no heterogeneity or pleiotropy in the sensitivity analysis of the forward and reverse MR analyses.

## Discussion

In this two-sample MR study, we investigated the potential causal relationship between genetically circulating VEGF levels and the risk of breast cancer, ovarian cancer, lung cancer, colorectal cancer, prostate cancer, esophageal cancer, kidney cancer, bladder cancer, thyroid cancer, malignant neoplasms of the brain, and malignant neoplasms of the liver and intrahepatic bile ducts. We found that genetically elevated concentrations of circulating VEGF were positively associated with the risk of colorectal cancer and colon adenocarcinoma, with suggestive evidence of association with rectal cancer but no evidence for reverse association.

Several previous observational studies have found that polymorphisms in VEGF may predispose patients to colorectal cancer susceptibility (Kim et al., 2008; Zhao et al., 2012; Jang et al., 2013; Espírito Santo et al., 2017; Yang et al., 2017). Jang et al. studied 882 participants in the Korean population, including 390 colorectal patients and 492 controls. The results showed that VEGF 936 C > T polymorphisms may contribute to colorectal cancer risk, and Haplotype-2578A/-1154A/-634G/936T of VEGF polymorphisms in the haplotype analyses were associated with an increased susceptibility to colorectal cancer. A meta-analysis of 12 epidemiological studies included 2770 colon cancer cases and 2568 controls. The results suggested that VEGF-460T/C, -634 g/C, and -2578C/A gene polymorphisms are associated with colorectal cancer risk (Zhao et al., 2012). In addition, another study explored the relationship between VEGF polymorphisms and rectal cancer and colon cancer. The results showed that VEGF 1451 C > T was significantly associated with rectal cancer risk, and VEGF 1725 G >A was associated with elevated colon cancer risk (Jeon et al., 2014). In our study, we used publicly available GWAS summary statistics, selected polymorphisms closely associated with circulating VEGF as exposure, and applied MR analysis to divide VEGF into high and low groups at the gene level to investigate the causal relationship between VEGF and the risk of various cancers. Similar to the above observational studies, we found that high levels of circulating VEGF increased the risk of colorectal cancer, colon cancer, and rectal cancer.

Angiogenesis inhibition is now well established for blocking tumor angiogenesis and targeting vascular endothelial cells. Angiogenesis can be targeted through a variety of mechanisms: binding to angiogenic factors, blocking angiogenic factor receptors, interrupting intracellular signaling pathways, and mimicking endogenous angiogenic inhibitors (Sivakumar et al., 2004). The anti-vascular endothelial growth factor antibody bevacizumab has been approved as a standard treatment for metastatic colorectal cancer in combination with chemotherapy. Bevacizumab binds to VEGF and prevents it from binding to receptors on the surface of endothelial cells. In addition to first-line therapy for metastatic colon cancer, the ML18147 study demonstrated clinical benefit in patients with metastatic colorectal cancer by maintaining a vascular endothelial growth factor inhibitor (bevacizumab) with standard second-line chemotherapy after disease progression (Bennouna et al., 2013). In the BRiTE study, the median overall survival was 31.8 months for patients who continued bevacizumab after disease progression compared with 19.9 months for patients who did not continue bevacizumab (Grothey et al., 2008). In the ARIES study, first-progression survival was 14.1 months for patients who continued bevacizumab after disease progression compared with 7.5 months for patients who did not receive bevacizumab (Bendell et al., 2012). Bevacizumab plays an important role in the treatment of colorectal cancer upon combination with VEGF. The conclusion of our study also plays an indirect role in confirming this relationship. Since the increase in VEGF is the cause of colorectal cancer, the combination and reduction of VEGF can improve the prognosis of colorectal cancer patients.

Several clinical observational studies have found that the circulating VEGF level in colorectal cancer patients is significantly higher than that in healthy people, and its level is related to pathological stage, lymph node or distant metastasis, and overall survival rate (Fujisaki et al., 1998; Cubo et al., 2004; Kwon et al., 2010; Bendardaf et al., 2017). One study measured preoperative serum VEGF in 35 patients with colorectal cancer and 30 healthy controls. Serum VEGF was higher in colorectal cancer patients with and without metastasis than in healthy controls. In addition, VEGF was found to be significantly higher in patients with advanced clinical stage disease than in patients with early clinical stage disease and in patients with metastases than in those with local lesions. The diagnostic accuracy of VEGF invasiveness was 83%, the sensitivity was 79%, and the specificity was 68% (Eldesoky et al., 2011). Elevated circulating VEGF is often detected in colorectal cancer, but our results show that colorectal cancer is not the cause of elevated circulating VEGF. In addition to colorectal cancer, no causal relationship with VEGF was found in any other tumor types. However, in MR analysis of the causal relationship between different tumor types and VEGF, the number of SNPs strongly associated with multiple tumor types was relatively small, except for breast cancer. Invalid results may have been due to low power and insufficient SNPs, which limited our ability to draw true causal conclusions.

VEGF polymorphisms have also been studied in other tumors. A cohort study of Tunisian women found that specific VEGF variants (RS699947, RS1570360) and haplotypes (CTGCCAG) may contribute to the development of cervical cancer (Zidi et al., 2015). VEGF polymorphisms have also been found to be significantly associated with susceptibility and aggressiveness of breast cancer (Rahoui et al., 2014; Sa-Nguanraksa et al., 2014), as well as prognosis of non-small-cell lung cancer (Heist et al., 2008), gastric cancer (Tzanakis et al., 2006) and esophageal cancer (Bradbury et al., 2009). However, in our MR analysis, no causal relationship was found between circulating VEGF and other types of cancer except colorectal cancer, colon cancer and rectal cancer. In observational studies, the number of VEGF gene polymorphisms included in the study was small, basically no more than 3, which made the inferred conclusions less effective. In our study, the number of gene polymorphisms closely associated with VEGF was 11, and the F-statistic of each SNP was over 10, suggesting that the bias due to measurement error was not more than 10% of the true value of the causal effect. I^2^ values were all close to 1, which indicated that the weak instrument bias was very small. Unlike observational studies, reverse causality and the bias introduced by cofounders can be avoided by using Mendelian random assignment as instrumental variables. In addition, sensitivity analysis was performed using a variety of methods to ensure the validity of causal conclusions.

However, some limitations of MR analysis that should be paid attention to. The outstanding problem is how to avoid pleiotropy of SNPs selected as instrumental variables. If SNPs affect multiple outcomes through independent factors, it is difficult to prove that the inference of exposure to outcome is not biased. Usually MR-Egger intercept and MR-PRESSO methods are used to detect horizontal pleiotropy in order to reduce bias. MR-PRESSO is unable to completely remove pleiotropy SNPs for unmeasured confounders, and MR-Egger often suffers from large estimation errors and thus has low power to detect the causal effect. MR methods using genome-wide summary statistics (Morrison et al., 2020) may relieve the concern for possibly false positive causal findings. No heterogeneity and horizontal pleiotropy were found in the MR analysis results in our study, which also proves the robustness of the results. In addition, we only used the full summary statistics of VEGF in the three loci identified from the circulating cytokines GWAS, the results might be underpowered. The small number of SNPs strongly associated with some tumor types in the reverse MR analysis may limit the ability to draw conclusions about causal relationship. The invalid results may be related to low power and insufficient SNP. Third, only participants of European ancestry were included in this study; thus, this conclusion can only be applied to European ancestry, and further validation is required for other ancestry populations. There is a possible biased estimate when the MR was conducated in the same population (i.e, Finnish Europeans). In addition to the effects of population race, inferences about causality can also be confounded by different population structures.

## Conclusion

In 24 types of cancer, circulating VEGF had a potential causal relationship with colorectal cancer and colon adenocarcinoma, and suggestive evidence of association had been found in rectal cancer. However, the causal relationship of VEGF on other types of cancer and reverse association was not identified. This finding indicates the importance of circulating VEGF in the prevention and treatment of colorectal cancer.

## Data Availability

GWAS summary statistics for circulating VEGF can be downloaded from Ahola-Olli’s meta-analysis(Ahola-Olli et al., 2017). GWAS summary statistics for breast cancer can be downloaded from the BCAC consortium website (http://bcac.ccge.medschl.cam.ac.uk/bcacdata/). GWAS summary statistics for ovarian cancer can be downloaded from the OCAC consortium website (http://ocac.ccge.medschl.cam.ac.uk/dataprojects/). GWAS summary statistics for lung cancer from the ILCCO consortium is publicly available. GWAS summary statistics for colorectal cancer, anus and anal canal cancer, prostate cancer, esophageal cancer, kidney cancer, bladder cancer, thyroid cancer, malignant neoplasm of brain and malignant neoplasm of liver and intrahepatic bile ducts can be downloaded from the FinnGen consortium website (https://finngen.gitbook.io/ documentation/datadownload). GWAS summary statistics for the above mentioned cancers can also be accessed from MR-Base database (http://app.mrbase.org/).Additional GWAS statistics for VEGF (Folkersen et al, 2020) and colorectal cancer (Huyghe et al, 2019) are used in the validation phase.

## Supplementary material

Supplementary Figure S1 Scatter plot of the causal effect of circulating VEGF on different types of cancer. (**A**) Breast cancer; (**B**) ER+ Breast cancer; (**C**) ER- Breast cancer; (**D**) Ovarian cancer; (**E**) High grade serous ovarian cancer; (**F**) Low grade serous ovarian cancer; (**G**) Invasive mucinous ovarian cancer; (**H**) Clear cell ovarian cancer; (**I**) Endometrioid ovarian cancer; (**J**) High grade and low grade serous ovarian cancer; (**K**) Lung cancer; (**L**) Lung adenocarcinoma; (**M**) Squamous cell lung cancer; (**N**) Colorectal cancer; (**O**) Colon adenocarcinoma; (**P**) Malignant neoplasm of rectum; (**Q**) Malignant neoplasm of anus and anal canal; (**R**) Malignant neoplasm of prostate; (**S**) Malignant neoplasm of oesophagus; (**T**) Malignant neoplasm of kidney, except renal pelvis; (**U**) Malignant neoplasm of bladder; (**V**) Malignant neoplasm of thyroid gland; (**W**) Malignant neoplasm of brain; (**X**) Malignant neoplasm of liver and intrahepatic bile ducts.

Supplementary Figure S2 Funnel plot of the causal effect of circulating VEGF on different types of cancer. (**A**) Breast cancer; (**B**) ER+ Breast cancer; (**C**) ER- Breast cancer; (**D**) Ovarian cancer; (**E**) High grade serous ovarian cancer; (**F**) Low grade serous ovarian cancer; (**G**) Invasive mucinous ovarian cancer; (**H**) Clear cell ovarian cancer; (**I**) Endometrioid ovarian cancer; (**J**) High grade and low grade serous ovarian cancer; (**K**) Lung cancer; (**L**) Lung adenocarcinoma; (**M**) Squamous cell lung cancer; (**N**) Colorectal cancer; (**O**) Colon adenocarcinoma; (**P**) Malignant neoplasm of rectum; (**Q**) Malignant neoplasm of anus and anal canal; (**R**) Malignant neoplasm of prostate; (**S**) Malignant neoplasm of oesophagus; (**T**) Malignant neoplasm of kidney, except renal pelvis; (**U**) Malignant neoplasm of bladder; (**V**) Malignant neoplasm of thyroid gland; (**W**) Malignant neoplasm of brain; (**X**) Malignant neoplasm of liver and intrahepatic bile ducts.

Supplementary Figure S3 Leave-one-out inverse-variance weighted mendelian randomization-analyses of circulating VEGF on different types of cancer. (**A**) Breast cancer; (**B**) ER+ Breast cancer; (**C**) ER- Breast cancer; (**D**) Ovarian cancer; (**E**) High grade serous ovarian cancer; (**F**) Low grade serous ovarian cancer; (**G**) Invasive mucinous ovarian cancer; (**H**) Clear cell ovarian cancer; (**I**) Endometrioid ovarian cancer; (**J**) High grade and low grade serous ovarian cancer; (**K**) Lung cancer; (**L**) Lung adenocarcinoma; (**M**) Squamous cell lung cancer; (**N**) Colorectal cancer; (**O**) Colon adenocarcinoma; (**P**) Malignant neoplasm of rectum; (**Q**) Malignant neoplasm of anus and anal canal; (**R**) Malignant neoplasm of prostate; (**S**) Malignant neoplasm of oesophagus; (**T**) Malignant neoplasm of kidney, except renal pelvis; (**U**) Malignant neoplasm of bladder; (**V**) Malignant neoplasm of thyroid gland; (**W**) Malignant neoplasm of brain; (**X**) Malignant neoplasm of liver and intrahepatic bile ducts.

Supplementary Figure S4 Scatter plot of the causal effect of different types of cancer on circulating VEGF. (**A**) Breast cancer; (**B**) ER+ Breast cancer; (**C**) ER- Breast cancer; (**D**) Ovarian cancer; (**E**) High grade serous ovarian cancer; (**F**) Invasive mucinous ovarian cancer; (**G**) High grade and low grade serous ovarian cancer; (**H**) Lung cancer; (**I**) Lung adenocarcinoma; (**J**) Squamous cell lung cancer; (**K**) Colorectal cancer; (**L**) Malignant neoplasm of prostate; (**M**) Malignant neoplasm of bladder; (**N**) Malignant neoplasm of thyroid gland.

Supplementary Figure S5 Funnel plot of the causal effect of different types of cancer on circulating VEGF. (**A**) Breast cancer; (**B**) ER+ Breast cancer; (**C**) ER- Breast cancer; (**D**) Ovarian cancer; (**E**) High grade serous ovarian cancer; (**F**) Invasive mucinous ovarian cancer; (**G**) High grade and low grade serous ovarian cancer; (**H**) Lung cancer; (**I**) Lung adenocarcinoma; (**J**) Squamous cell lung cancer; (**K**) Malignant neoplasm of prostate.

Supplementary Figure S6 Leave-one-out inverse-variance weighted mendelian randomization-analyses of different types of cancer on circulating VEGF. (**A**) Breast cancer; (**B**) ER+ Breast cancer; (**C**) ER- Breast cancer; (**D**) Ovarian cancer; (**E**) High grade serous ovarian cancer; (**F**) Invasive mucinous ovarian cancer; (**G**) High grade and low grade serous ovarian cancer; (**H**) Lung cancer; (**I**) Lung adenocarcinoma; (**J**) Squamous cell lung cancer; (**K**) Colorectal cancer.
