# Supplementary figures and images for: Circulating vascular endothelial growth factor and cancer risk: A bidirectional mendelian randomization
Source: Front Genet. 2022 Sep 7;13:981032. doi: 10.3389/fgene.2022.981032 (PMC9489904; doi:10.3389/fgene.2022.981032)

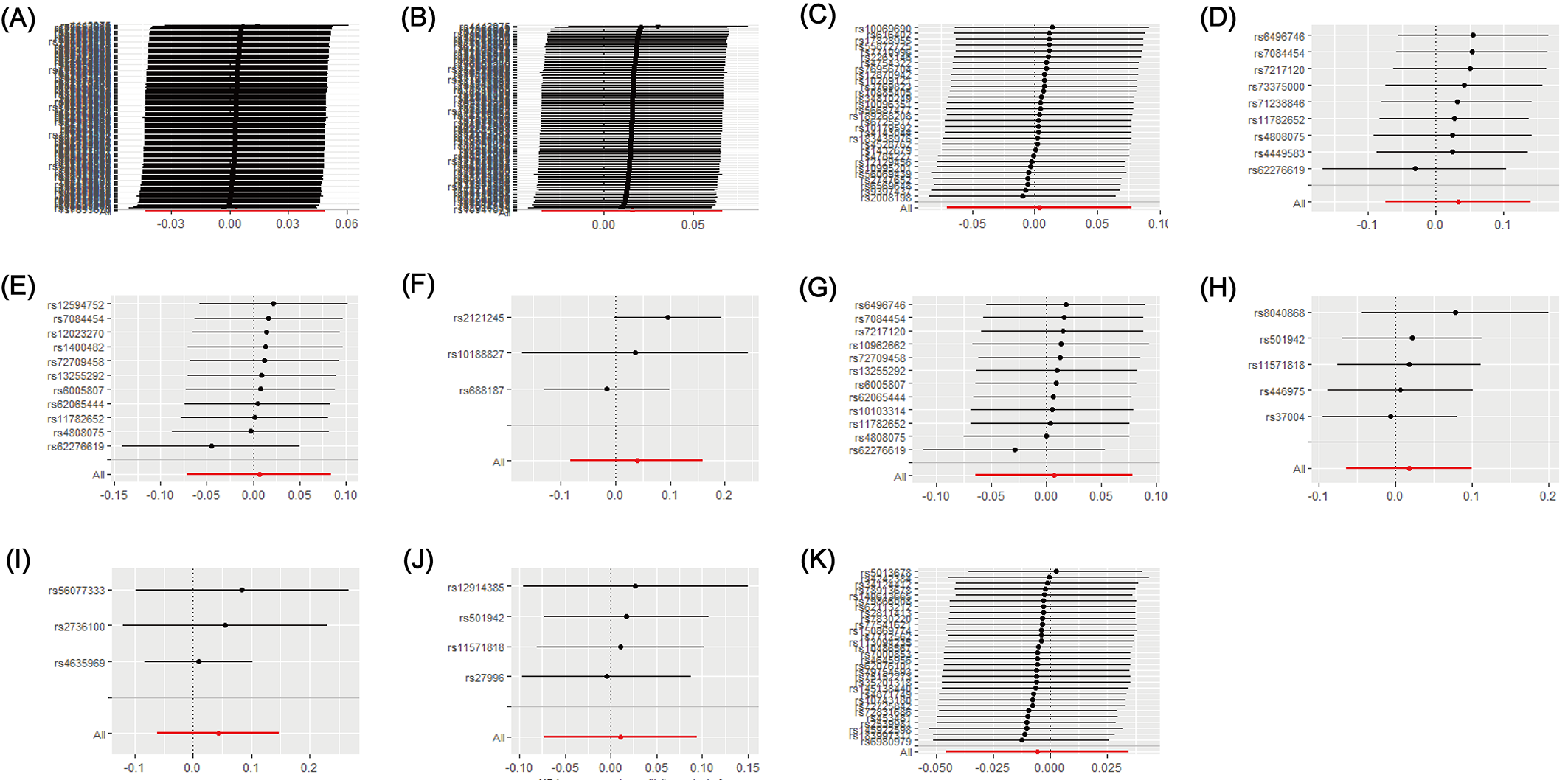

Supplement: Supplementary file 1 [file Image6.TIF]

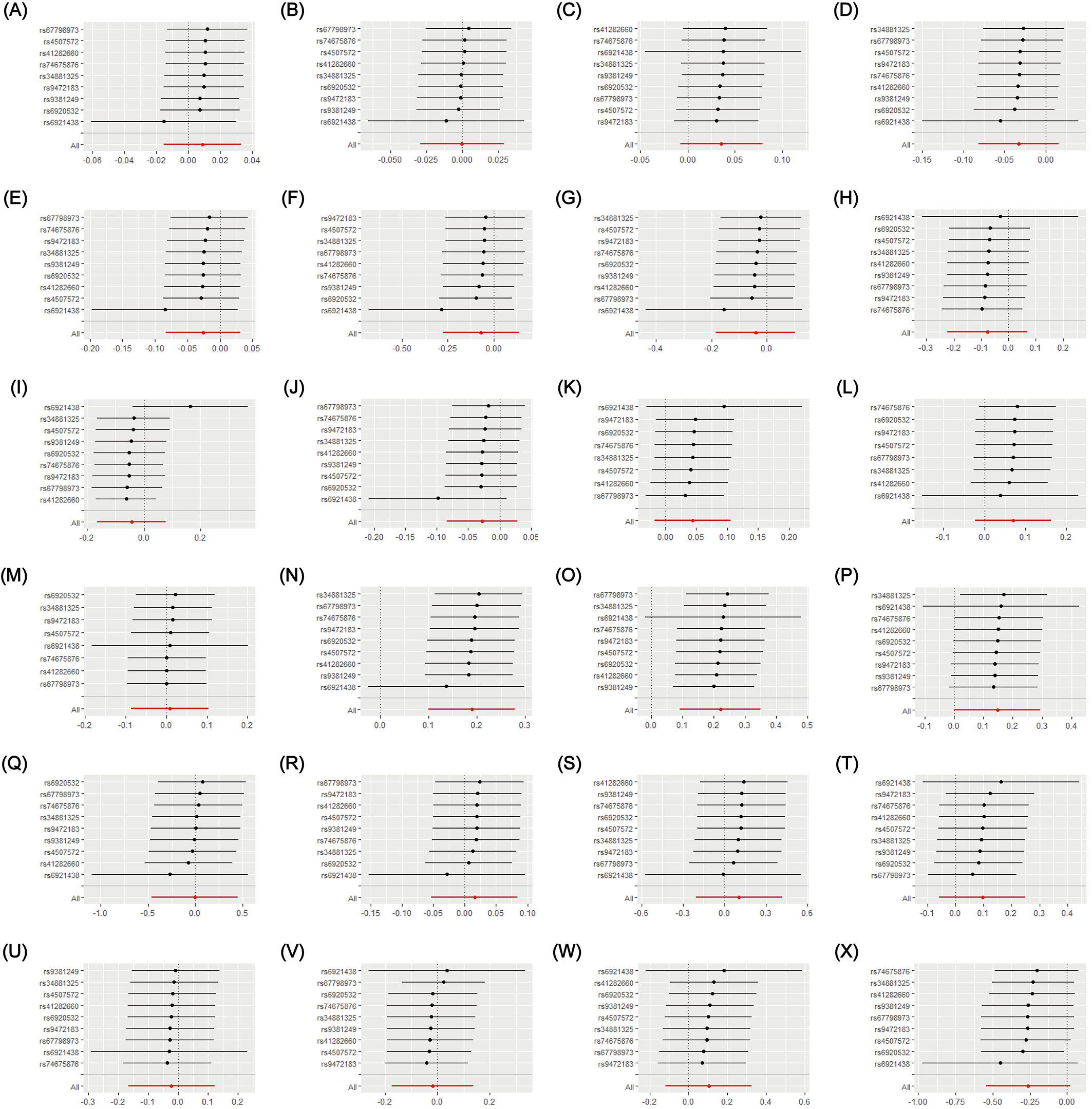

Supplement: Supplementary file 3 [file Image3.TIF]

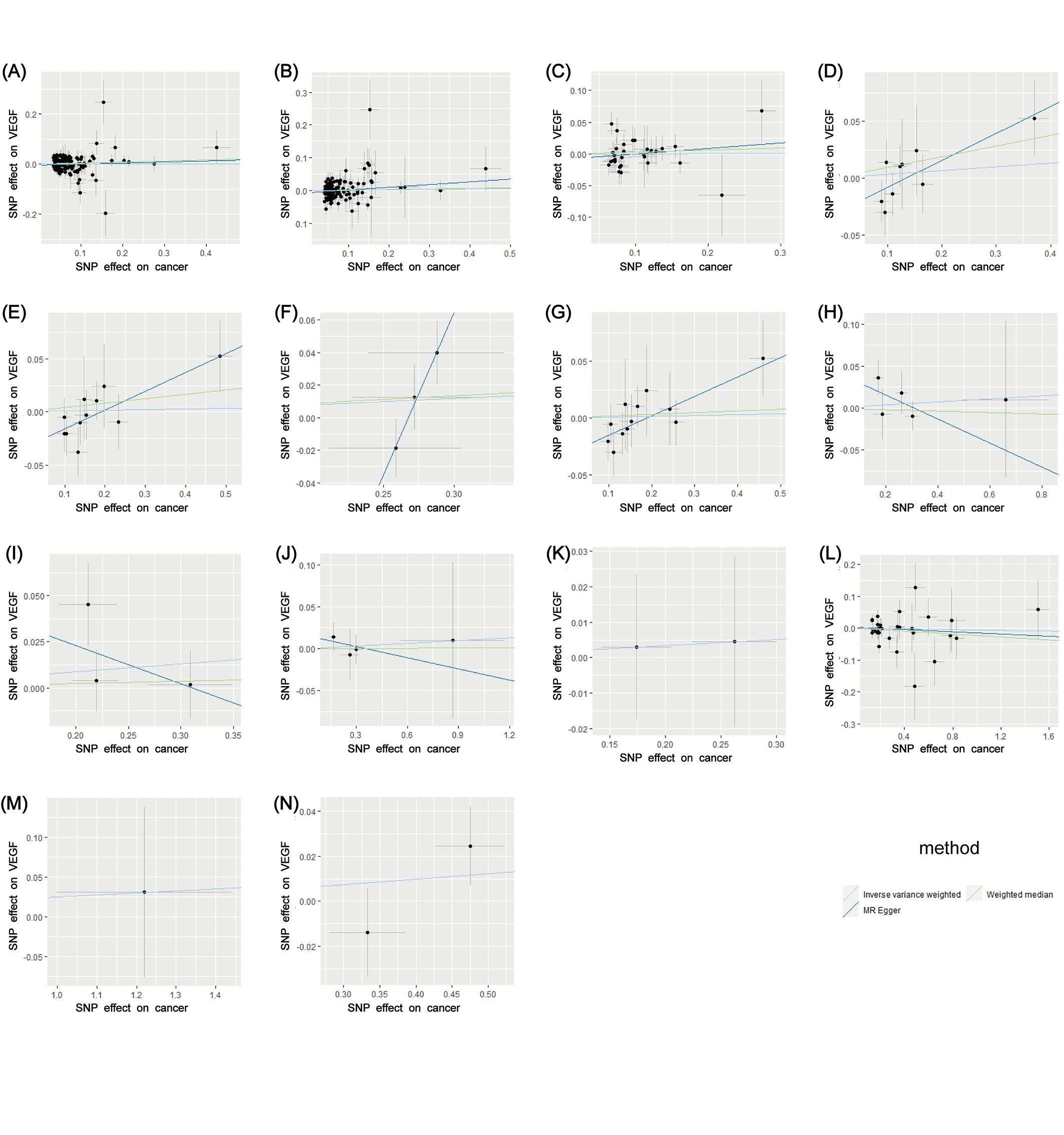

Supplement: Supplementary file 4 [file Image4.TIF]

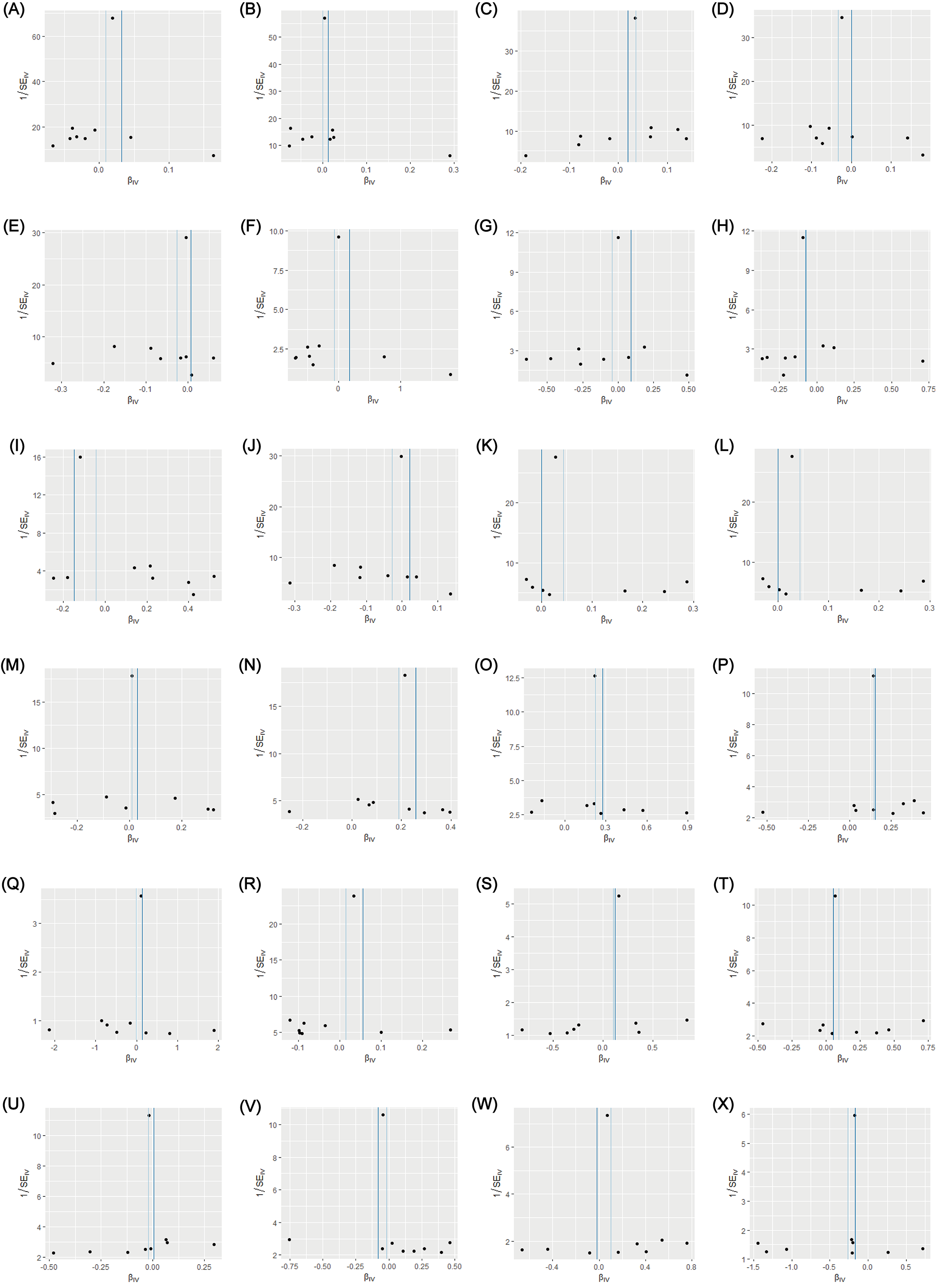

Supplement: Supplementary file 5 [file Image2.TIF]

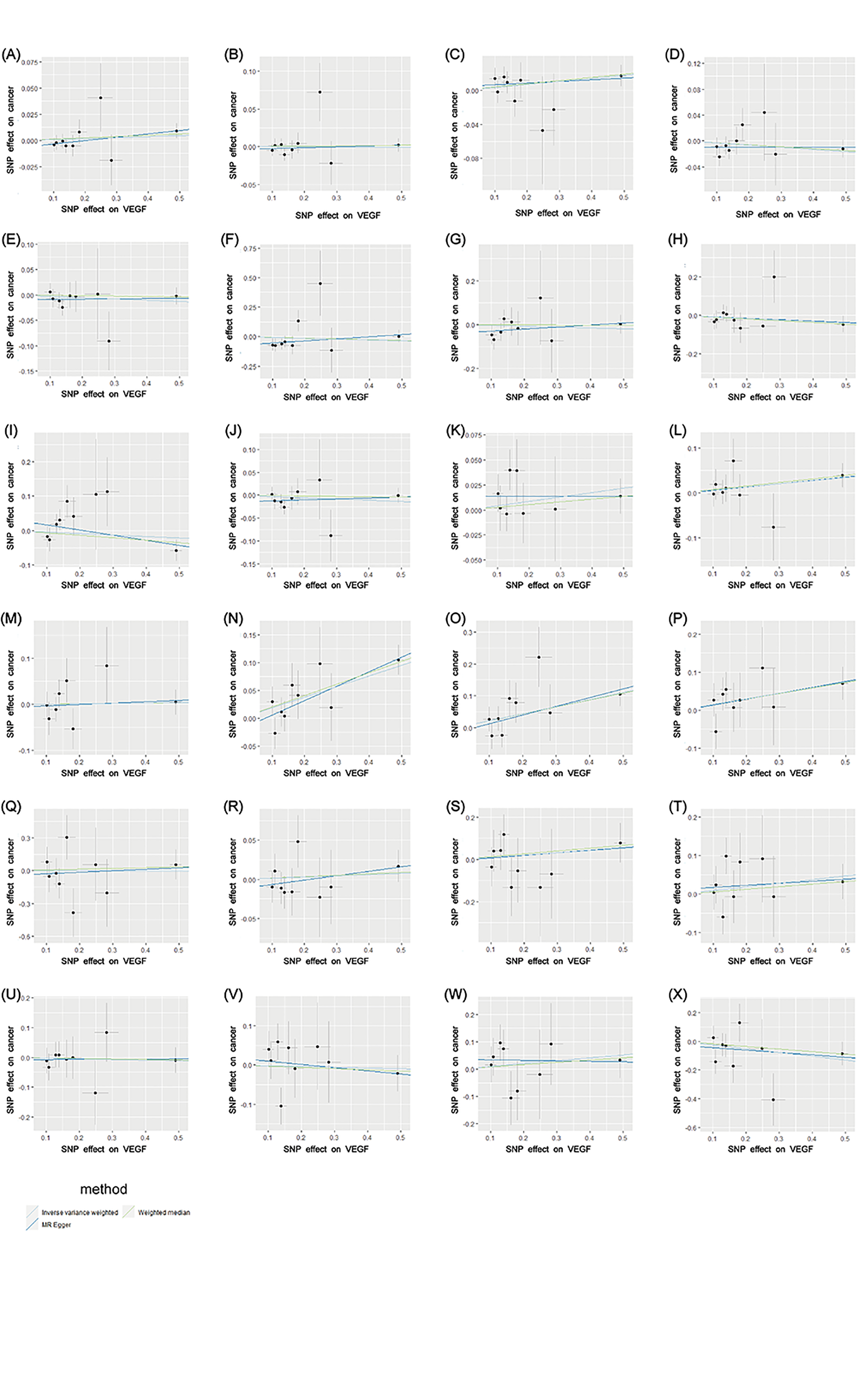

Supplement: Supplementary file 6 [file Image1.TIF]

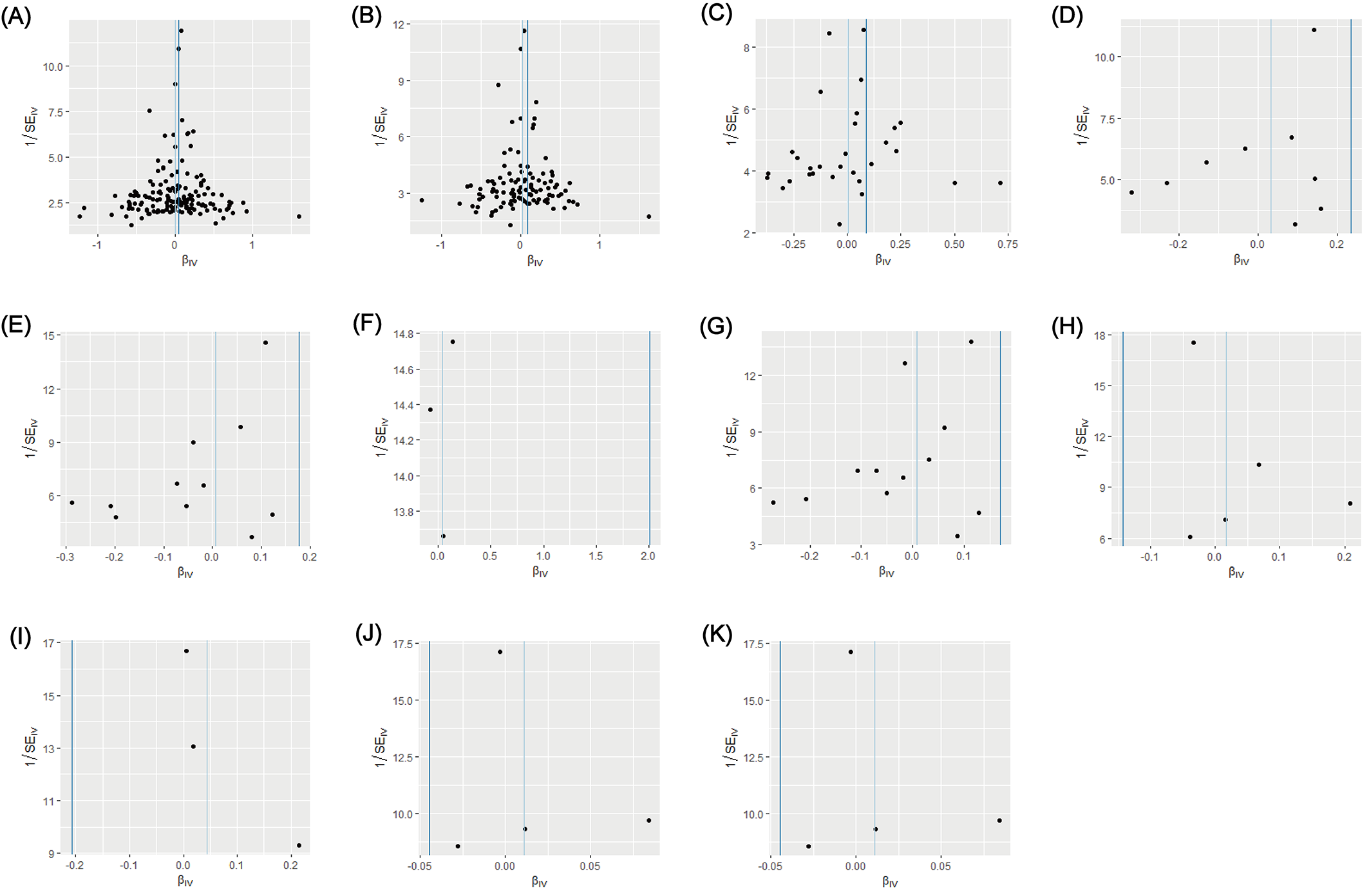

Supplement: Supplementary file 7 [file Image5.TIF]
