# Supplementary material for: Circulating vascular endothelial growth factor and cancer risk: A bidirectional mendelian randomization
Source: Front Genet. 2022 Sep 7;13:981032. doi: 10.3389/fgene.2022.981032 (PMC9489904; doi:10.3389/fgene.2022.981032)
Supplement: Supplementary file 2 [file Table1.DOCX]

**Table S1. Summary of data source of different types of cancer**

|  | Consortium | Number of SNPs | Ncases | Ncontrols | Sample size | | Year | Population | Sex |
| --- | --- | --- | --- | --- | --- | --- | --- | --- | --- |
| Breast Cancer | BCAC | 10,680,257 | 122,977 | \| 105,974 \| \| --- \| | 228,951 | \| 2017 \| \| --- \| | | European | Females |
| ER+ Breast cancer | BCAC | 10,680,257 | 69,501 | \| 105,974 \| \| --- \| | 175,475 | \| 2017 \| \| --- \| | | European | Females |
| ER- Breast cancer | BCAC | 10,680,257 | 21,468 | \| 105,974 \| \| --- \| | 127,442 | \| 2017 \| \| --- \| | | European | Females |
| Ovarian cancer | OCAC | 11403952 | 25,509 | \| 40,941 \| \| --- \| | 66,450 | \| 2017 \| \| --- \| | | European | Females |
| High grade serous ovarian cancer | OCAC | 11403952 | 13,037 | \| 40,941 \| \| --- \| | 53,978 | \| 2017 \| \| --- \| | | European | Females |
| Low grade serous ovarian cancer | OCAC | 11403952 | 1,012 | \| 40,941 \| \| --- \| | 41,953 | \| 2017 \| \| --- \| | | European | Females |
| Invasive mucinous ovarian cancer | OCAC | 11403952 | 1,417 | \| 40,941 \| \| --- \| | 42,358 | \| 2017 \| \| --- \| | | European | Females |
| Clear cell ovarian cancer | OCAC | 11403952 | 1,366 | \| 40,941 \| \| --- \| | 42,307 | \| 2017 \| \| --- \| | | European | Females |
| Endometrioid ovarian cancer | OCAC | 11403952 | 2,810 | \| 40,941 \| \| --- \| | 43,751 | \| 2017 \| \| --- \| | | European | Females |
| High grade and low grade serous ovarian cancer | OCAC | 11403952 | 14,049 | \| 40,941 \| \| --- \| | 54,990 | \| 2017 \| \| --- \| | | European | Females |
| lung cancer | ILCCO | 8,945,893 | 11,348 | \| 15,861 \| \| --- \| | 27,209 | \| 2014 \| \| --- \| | | European | Males and Females |
| Lung adenocarcinoma | ILCCO | 8,881,354 | 3,442 | \| 14,894 \| \| --- \| | 18,336 | \| 2014 \| \| --- \| | | European | Males and Females |
| Squamous cell lung cancer | ILCCO | 8,893,750 | 3,275 | 15,038 | 18,313 | 2014 | | European | Males and Females |
| Colorectal cancer | FinnGen | 16,380,466 | 3,022 | 215,770 | 218,792 | 2021 | | European | Males and Females |
| Colon adenocarcinoma | FinnGen | 16,380,466 | 1,396 | 217,396 | 218,792 | 2021 | | European | Males and Females |
| Malignant neoplasm of rectum | FinnGen | 16,380,466 | 1,078 | 217,714 | 218,792 | 2021 | | European | Males and Females |
| Malignant neoplasm of anus and anal canal | FinnGen | 16,380,466 | 108 | 218,684 | 218,792 | 2021 | | European | Males and Females |
| Malignant neoplasm of prostate | FinnGen | 16,378,835 | 6,311 | 88,902 | 218,792 | 2021 | | European | Males and Females |
| Malignant neoplasm of oesophagus | FinnGen | 16,380,466 | 232 | 218,560 | 218,792 | 2021 | | European | Males and Females |
| Malignant neoplasm of kidney, except renal pelvis | FinnGen | 16,380,466 | 971 | 217,821 | 218,792 | 2021 | | European | Males and Females |
| Malignant neoplasm of bladder | FinnGen | 16,380,466 | 1,115 | 217,677 | 218,792 | 2021 | | European | Males and Females |
| Malignant neoplasm of thyroid gland | FinnGen | 16,380,466 | 989 | 217,803 | 218,792 | 2021 | | European | Males and Females |
| Malignant neoplasm of brain | FinnGen | 16,380,466 | 464 | 218,328 | 218,792 | 2021 | | European | Males and Females |
| Malignant neoplasm of liver and intrahepatic bile ducts | FinnGen | 16,380,466 | 304 | 218,488 | 218,792 | 2021 | | European | Males and Females |

BCAC: Breast Cancer Association Consortium; OCAC: Ovarian Cancer Association Consortium; ILCCO: International Lung Cancer Consortium.

| SNP | Chr | Pos | EA | NEA | Beta | SE | P value | EAF | F | R^2^(%) |
| --- | --- | --- | --- | --- | --- | --- | --- | --- | --- | --- |
| rs9381249 | 6 | 43734798 | C | T | 0.248 | 0.040 | 3.085E-10 | 0.04 | 39.09 | 0.55 |
| rs6920532 | 6 | 43793430 | C | T | -0.180 | 0.027 | 8.676E-12 | 0.89 | 45.60 | 0.64 |
| rs67798973 | 6 | 43882777 | A | G | 0.139 | 0.017 | 1.289E-15 | 0.57 | 62.99 | 0.88 |
| rs6921438 | 6 | 43925607 | A | G | -0.490 | 0.018 | 2.089E-171 | 0.46 | 784.0 | 9.82 |
| rs9472183 | 6 | 43940202 | G | A | 0.128 | 0.017 | 5.186E-14 | 0.54 | 56.87 | 0.79 |
| rs74675876 | 6 | 43963995 | C | T | 0.282 | 0.037 | 7.619E-15 | 0.06 | 59.45 | 0.83 |
| rs4507572 | 6 | 44135095 | C | T | -0.101 | 0.017 | 3.342E-09 | 0.52 | 34.68 | 0.48 |
| rs41282660 | 6 | 44197006 | G | A | 0.161 | 0.026 | 1.328E-09 | 0.14 | 37.62 | 0.53 |
| rs34881325 | 9 | 2622134 | T | C | -0.108 | 0.019 | 1.044E-08 | 0.38 | 32.77 | 0.46 |
| rs7030781 | 9 | 2686273 | T | A | -0.137 | 0.017 | 2.565E-15 | 0.39 | 62.53 | 0.87 |
| rs10761731 | 10 | 65027610 | T | A | 0.119 | 0.017 | 1.006E-11 | 0.39 | 46.54 | 0.65 |

**Table S2: The characteristics of SNPs adopted as instrumental variables of circulating VEGF**

**Table S3. Mendelian randomization results of IVW, weighted median, and MR-Egger from circulating VEGF to** **different types of cancer.**

|  |  | IVW | | Weighted median | | MR-Egger | |  |  |  |
| --- | --- | --- | --- | --- | --- | --- | --- | --- | --- | --- |
|  | NSNP | OR (95% CI) | P | OR (95% CI) | P | OR (95% CI) | P | I^2^ | P_heterogeneity_ | P_pleiotropy_ |
| Breast Cancer | 9 | 1.01(0.98,1.03) | 0.47 | \| 1.01(0.99,1.04) \| \| --- \| | 0.37 | \| 1.03(0.99,1.08) \| \| --- \| | 0.18 | 0.98 | 0.86 | 0.22 |
| ER+ | 9 | 1.00(0.97,1.03) | 0.98 | \| 1.00(0.97,1.04) \| \| --- \| | 0.84 | \| 1.01(0.96,1.06) \| \| --- \| | 0.66 | 0.98 | 0.55 | 0.58 |
| ER- | 9 | 1.04(0.99,1.08) | 0.11 | \| 1.04(0.99,1.09) \| \| --- \| | 0.13 | \| 1.02(0.95,1.10) \| \| --- \| | 0.63 | 0.98 | 0.79 | 0.62 |
| Ovarian cancer | 9 | 0.97(0.92,1.02) | 0.19 | \| 0.97(0.92,1.03) \| \| --- \| | 0.29 | \| 1.00(0.92,1.09) \| \| --- \| | 0.99 | 0.98 | 0.81 | 0.36 |
| High grade serous ovarian cancer | 9 | 0.97(0.92,1.03) | 0.38 | \| 0.99(0.93,1.06) \| \| --- \| | 0.84 | \| 1.01(0.91,1.11) \| \| --- \| | 0.91 | 0.98 | 0.79 | 0.46 |
| Low grade serous ovarian cancer | 9 | 0.93(0.76,1.15) | 0.51 | \| 0.94(0.77,1.14) \| \| --- \| | 0.51 | \| 1.20(0.88,1.63) \| \| --- \| | 0.29 | 0.98 | 0.39 | 0.09 |
| Invasive mucinous ovarian cancer | 9 | 0.96(0.83,1.11) | 0.59 | \| 0.99(0.84,1.17) \| \| --- \| | 0.93 | \| 1.10(0.86,1.40) \| \| --- \| | 0.49 | 0.98 | 0.84 | 0.24 |
| Clear cell ovarian cancer | 9 | 0.93(0.80,1.07) | 0.30 | \| 0.91(0.77,1.07) \| \| --- \| | 0.27 | \| 0.93(0.72,1.19) \| \| --- \| | 0.59 | 0.98 | 0.77 | 0.96 |
| Endometrioid ovarian cancer | 9 | 0.96(0.85,1.08) | 0.49 | \| 0.93(0.83,1.05) \| \| --- \| | 0.24 | \| 0.86(0.71,1.05) \| \| --- \| | 0.19 | 0.98 | 0.28 | 0.24 |
| High grade and low grade serous ovarian cancer | 9 | 0.97(0.92,1.03) | 0.33 | \| 0.99(0.93,1.06) \| \| --- \| | 0.79 | \| 1.02(0.93,1.12) \| \| --- \| | 0.68 | 0.98 | 0.75 | 0.26 |
| lung cancer | 8 | 1.04(0.98,1.11) | 0.17 | \| 1.03(0.96,1.10) \| \| --- \| | 0.43 | \| 1.00(0.90,1.11) \| \| --- \| | 0.99 | 0.99 | 0.68 | 0.37 |
| Lung adenocarcinoma | 8 | 1.07(0.98,1.18) | 0.14 | \| 1.08(0.98,1.20) \| \| --- \| | 0.12 | \| 1.08(0.92,1.26) \| \| --- \| | 0.40 | 0.99 | 0.73 | 0.96 |
| Squamous cell lung cancer | 8 | 1.01(0.92,1.11) | 0.85 | 1.01(0.91,1.12) | 0.88 | 1.03(0.87,1.21) | 0.73 | 0.99 | 0.53 | 0.77 |
| Colorectal cancer | 9 | 1.21(1.11,1.32) | <0.00 | 1.23(1.11,1.36) | <0.00 | 1.30(1.11,1.51) | 0.01 | 0.98 | 0.71 | 0.33 |
| Colon adenocarcinoma | 9 | 1.25(1.10,1.42) | <0.00 | 1.24(1.08,1.43) | 0.00 | 1.32(1.04,1.67) | 0.06 | 0.98 | 0.37 | 0.60 |
| Malignant neoplasm of rectum | 9 | 1.16(1.00,1.34) | 0.049 | 1.16(0.98,1.37) | 0.09 | 1.17(1.90,1.51) | 0.27 | 0.98 | 0.77 | 0.94 |
| Malignant neoplasm of anus and anal canal | 9 | 0.10(0.63,1.57) | 0.99 | 1.07(0.64,1.81) | 0.79 | 1.14(0.51,2.54) | 0.75 | 0.98 | 0.43 | 0.69 |
| Malignant neoplasm of prostate | 9 | 1.02(0.95,1.09) | 0.66 | 1.02(0.94,1.10) | 0.64 | 1.06(0.94,1.19) | 0.39 | 0.98 | 0.80 | 0.44 |
| Malignant neoplasm of oesophagus | 9 | 1.11(0.81,1.52) | 0.50 | 1.14(0.81,1.62) | 0.45 | 1.13(0.65,1.95) | 0.68 | 0.98 | 0.81 | 0.95 |
| Malignant neoplasm of kidney, except renal pelvis | 9 | 1.10(0.94,1.28) | 0.22 | 1.07(0.90,1.27) | 0.46 | 1.06(0.80,1.39) | 0.71 | 0.98 | 0.42 | 0.72 |
| Malignant neoplasm of bladder | 9 | 0.98(0.85,1.13) | 0.77 | 0.98(0.84,1.15) | 0.83 | 1.00(0.78,1.29) | 0.97 | 0.98 | 0.92 | 0.81 |
| Malignant neoplasm of thyroid gland | 9 | 0.98(0.84,1.15) | 0.81 | 0.97(0.82,1.15) | 0.73 | 0.92(0.69,1.23) | 0.59 | 0.98 | 0.34 | 0.61 |
| Malignant neoplasm of brain | 9 | 1.11(0.89,1.39) | 0.36 | 1.09(0.84,1.41) | 0.53 | 0.98(0.66,1.45) | 0.93 | 0.98 | 0.69 | 0.47 |
| Malignant neoplasm of liver and intrahepatic bile ducts | 9 | 0.77(0.58,1.02) | 0.07 | 0.83(0.62,1.13) | 0.23 | 0.85(0.50,1.43) | 0.55 | 0.98 | 0.29 | 0.68 |

NSNP number of single nucleotide polymorphism, OR odds ratio, 95%CI lower and upper limit of 95% confidence interval, P p-value of OR, P_heterogeneity_ p-value of Cochrane’s Q value in heterogeneity test, P_pleiotropy_ p-value of MR-Egger intercept.

**Table S4. Mendelian randomization results of Wald ratio, IVW, weighted median, and MR-Egger from different types of cancer to circulating VEGF.**

|  |  | Wald ratio | | IVW |  | Weighted median | | MR-Egger | |  |  |
| --- | --- | --- | --- | --- | --- | --- | --- | --- | --- | --- | --- |
|  | NSNP | OR (95% CI) | P | OR (95% CI) | P | OR (95% CI) | P | OR (95% CI) | P | P_heterogeneity_ | P_pleiotropy_ |
| Breast Cancer | 156 | NA | NA | 1.00(0.96,1.05) | 0.90 | \| 1.04(0.96,1.13) \| \| --- \| | 0.31 | \| 1.04(0.95,1.15) \| \| --- \| | 0.39 | 0.63 | 0.36 |
| ER+ | 115 | NA | NA | 1.02(0.97,1.07) | 0.55 | \| 1.02(0.94,1.10) \| \| --- \| | 0.67 | \| 1.09(0.97,1.21) \| \| --- \| | 0.14 | 0.20 | 0.18 |
| ER- | 30 | NA | NA | 1.00(0.93,1.08) | 0.92 | \| 1.03(0.93,1.15) \| \| --- \| | 0.57 | \| 1.09(0.87,1.37) \| \| --- \| | 0.46 | 0.52 | 0.45 |
| Ovarian cancer | 9 | NA | NA | 1.03(0.93,1.15) | 0.55 | \| 1.10(0.96,1.26) \| \| --- \| | 0.18 | \| 1.27(0.99,1.61) \| \| --- \| | 0.10 | 0.79 | 0.11 |
| High grade serous ovarian cancer | 11 | NA | NA | 1.01(0.93,1.09) | 0.87 | \| 1.04(0.94,1.16) \| \| --- \| | 0.45 | \| 1.19(1.01,1.41) \| \| --- \| | 0.07 | 0.95 | 0.05 |
| Invasive mucinous ovarian cancer | 3 | NA | NA | 1.04(0.92,1.17) | 0.53 | \| 1.05(0.93,1.17) \| \| --- \| | 0.45 | \| 7.46(1.24,44.8) \| \| --- \| | 0.27 | 0.85 | 0.28 |
| High grade and low grade serous ovarian cancer | 12 | NA | NA | 1.01(0.94,1.08) | 0.84 | \| 1.01(0.92,1.12) \| \| --- \| | 0.77 | \| 1.19(1.01,1.40) \| \| --- \| | 0.07 | 0.98 | 0.06 |
| lung cancer | 5 | NA | NA | 1.02(0.94,1.10) | 0.67 | \| 0.99(0.90,1.09) \| \| --- \| | 0.86 | \| 0.87(0.65,1.16) \| \| --- \| | 0.40 | 0.52 | 0.34 |
| Lung adenocarcinoma | 3 | NA | NA | 1.04(0.94,1.16) | 0.42 | \| 1.01(0.92,1.11) \| \| --- \| | 0.80 | \| 0.81(0.41,1.61) \| \| --- \| | 0.66 | 0.16 | 0.60 |
| Squamous cell lung cancer | 4 | NA | NA | 1.01(0.93,1.10) | 0.80 | 1.00(0.91,1.10) | 0.99 | 0.96(0.76,1.20) | 0.74 | 0.81 | 0.66 |
| Colorectal cancer | 2 | NA | NA | 1.02(0.88,1.17) | 0.81 | NA | NA | NA | NA | 1.00 | NA |
| Malignant neoplasm of prostate | 30 | NA | NA | 0.99(0.96,1.03) | 0.79 | 0.98(0.93,1.03) | 0.40 | 0.98(0.91,1.06) | 0.65 | 0.25 | 0.71 |
| Malignant neoplasm of bladder | 1 | 1.03(0.86,1.22) | 0.77 | NA | NA | NA | NA | NA | NA | NA | NA |
| Malignant neoplasm of thyroid gland | 2 | NA | NA | 1.02(0.94,1.11) | 0.56 | NA | NA | NA | NA | 0.18 | NA |
| Malignant neoplasm of liver and intrahepatic bile ducts | 1 | 1.01(0.94,1.08) | 0.83 | NA | NA | NA | NA | NA | NA | NA | NA |

NSNP number of single nucleotide polymorphism, OR odds ratio, 95%CI lower and upper limit of 95% confidence interval, P p-value of OR, Pheterogeneity p-value of Cochrane’s Q value in heterogeneity test, Ppleiotropy p-value of MR-Egger intercept.
